# Complete Replacement of Nitrite With a *Lactobacillus fermentum* on the Quality and Safety of Chinese Fermented Sausages

**DOI:** 10.3389/fmicb.2021.704302

**Published:** 2021-08-04

**Authors:** Yuning Xu, Yinglian Zhu

**Affiliations:** ^1^College of Food Science and Engineering, Qingdao Agricultural University, Qingdao, China; ^2^Qingdao Special Food Research Institute, Qingdao, China

**Keywords:** nitrite, *Lactobacillus fermentum*, fermented sausages, biological amines, thiobarbituric acid reactive substances, total volatile basenitrogen, metmyoglobin

## Abstract

This study investigated the positive effects of complete replacement of nitrite with a *Lactobacillus fermentum* on the quality and safety of Chinese fermented sausages, and evaluated the risk of this strain. The effects of the strain on pH, color, nitrite, thiobarbituric acid reactive substances (TBARS), total volatile basenitrogen (TVB-N), metmyoglobin (Met-Mb), biological amines, free amino acid content, and sensory index have been studied. The results revealed that the strain reduced the pH of the sausages, which reduced the risk of food-borne pathogens, and accelerated the acidification and gelation process. The inoculation of the strain produced pink color similar to 50 mg/kg nitrite, significantly reducing the residual risk of nitrite in the sausages. In addition, the strain effectively improved quality and nutrition of the sausages through preventing fat oxidation, protein decomposition, and myoglobin oxidation and increasing free amino acid content. The harmful biogenic amines species of the treated sample were reduced, although the tyramine contents were higher than the control, and the contents of the two groups were all far below the specified limit (800 mg/kg). The sensory analysis showed that the strain enhanced the taste, flavor, sourness, and overall acceptability of the sample sausages. Therefore, replacing nitrite completely with the strain *L. fermentum* could be a potential strategy to produce healthier and safer acceptable sausages through decreasing the risk of nitrite and improving nutrition and quality of the sausages.

## Introduction

In food process, specific food additives are often added to prevent corruption and extend the shelf life of the foods. However, the residual of some chemical additives will threaten human health, and they might also form toxic compounds due to the chemical reaction between the reactants (Molognoni et al., 2019). Hence, the development of natural and harmless food additives has become research hotspots in food industry. The natural biopreservatives are ideal natural food preservatives, which are safe, nontoxic, and efficient and have no negative effects. However, there are fewer natural microbial preservatives that can be used in the meat industry, and in-depth explorations are needed.

Chinese fermented sausages have unique flavor and rich nutrition, so they are very popular among people. However, naturally fermented sausages are easily contaminated by bacteria and have a short shelf life. Usually, certain food additives are added in the process of naturally fermented sausages to extend the shelf life. Among them, nitrite is often used in the process because it has color development ability, antioxidant activity, and antibacterial effects. However, the products of protein degradation during meat storage would react with nitrite to produce N-nitrosamine (Drabik-Markiewicz et al., 2011), which has carcinogenic effects to humans. As people pay more and more attention to health, it is very urgent to develop natural preservatives that are harmless to human health in the meat industry.

Lactic acid bacteria (LAB), as a kind of probiotics, are often used as starters for foods, such as sausages and yogurts. A previous report indicated that the LAB inoculated in the meat products could degrade nitrite and scavenge free radicals, and exhibited antioxidant activity and antibacterial effects (Ren et al., 2014). A previous report indicated that the *Lactobacillus plantarum* P2 isolated from traditional fermented sauerkraut had the ability to scavenge hydroxyl free radicals and superoxide free radicals and could degradate nitrite effectively (Chen et al., 2019). Zhang et al. (2020) found that the *L. plantarum* LPL-1 inhibited the growth of spoilage bacteria during fermentation and significantly reduced the content of histamine, putrescine, and cadaverine, and the total amount of biogenic amines of sausages. Guimarães et al. (2018) showed that the *L. plantarum* UM55 CFS could produce lactic acid along with other organic acids during fermentation, which has been confirmed to inhibit the growth of *Aspergillus flavus*. In addition, the *Lactobacillus fermentum* R6 was found to have antibacterial ability against the growth of *Clostridium perfringens* and its spores in chicken meat, so the strain could be used as a potential biopreservatives to prevent the contamination by *C. perfringens* in meat products (Li et al., 2017).

Some other studies pointed that the LAB had the effect of improving color in fermented sausages. The effect of different concentrations of *L. fermentum* on the color development of fermented sausages was studied, and the results showed that the strain contributed to the production of nitrosomyoglobin (Mb-NO) and 10^8^ CFU/g meat of the strain could produce the pink color similar to 60 mg/kg nitrite (Zhang et al., 2007). The research of Kawahara et al. (2006) showed that *Lactobacillus sakei* M32 could produce the red color with high redness (*a*^∗^ value) and low yellowness (*b*^∗^ value), and the color of the sample was similar to that of the control with nitrite added. Chen et al. (2016) isolated two strains of *L. plantarum* CMRC6 and *L. sakei* CMRC15 from traditional fermented pork and found that the two strains promoted nitrosation of myoglobin and produced the pink color in the sausages due to their nitrite reductase activity. In addition, LAB is usually used to improve the flavor and texture of fermented foods as a starter due to its acid production capacity. Moreover, LAB have many other functions, including regulating the intestinal flora, improving immunity, lowering cholesterol, etc. (Khalique et al., 2020; Saravanakumar et al., 2020). Therefore, the inoculation of LAB in foods has great value to improve food nutrition and protect human health (Sharafedtinov et al., 2013; Tu et al., 2018).

The above reports indicated that LAB have color development and antibacterial activity, so they might have the potential to replace nitrite in meat processing. However, no practical LAB strains have yet been found for industrial application as a complete substitute for nitrite. Therefore, the replacement for nitrite with LAB in meat products still requires in-depth exploration. The target of this study was to evaluate the positive effects of complete replacement of nitrite with the *L. fermentum* (CICC 21828) on the quality and safety of Chinese fermented sausages. The effects of the strain on pH, color, nitrite, thiobarbituric acid reactive substances (TBARS), total volatile basenitrogen (TVB-N), metmyoglobin (Met-Mb), tyramine, free amino acid content, and sensory index have been studied.

## Materials and Methods

### Strains

The LAB used in this experiment were *L. fermentum* (CICC 21828), which was provided by China Center of Industrial Culture Collection and preserved in the fermentation engineering laboratory, Qingdao Agricultural University.

### Sausage Manufacture

The formulation of the sausages includes 75% lean pork meat, 25% pork back fat, 2.3% NaCl, 3% sucrose, and 1% D-sodium erythorbate. The pork and pork casings were obtained from a local retailer in Qingdao (China). In addition, the 0.005% (50 mg/kg) NaNO_2_ was added in the control (Yoo et al., 2015), and 10^10^ CFU/g meat of the strain *L. fermentum* was inoculated in the sample to substrate 50 mg/kg NaNO_2_. All the raw materials were chopped, mixed, and marinated at 4°C for 24 h. The mixture was then stuffed in natural pork casings, fermented at 37°C in an incubator for 3 h with relative humidity (RH) of 90%, and then hung and dehydrated at 10°C for 20 days, with an RH of 70% according to our previous report (Zhu et al., 2020). Finally, the sausages were baked at 80°C for 1 h, at 65°C for 8 h, and then steamed at 100°C for 30 min.

### pH and Chroma

The sausages were minced to small pieces and were homogenized in 90 ml of sterilized saline (0.85%, w/v) in a BagMixer for 90 s, and then the pH was detected using a pH meter (FiveEasy Plus 28, Mettler Toledo, Shanghai, China) with a solid electrode. The sausages were sliced into slices about 2 cm and wrapped with a layer of film, and the chroma measurement was carried out with a Chroma Meter (CR-400, Konica Minolta Co., Tokyo, Japan). The values of lightness (*L*^∗^ value), *a*^∗^ value, and *b*^∗^ value were recorded from different pieces of each sample.

### Nitrite Residual

The nitrite residual was measured with the method of hydrochloride naphthodiamide according to a previous report (Zhang et al., 2007) with minor modification. The sausages were minced to small pieces and were pounded with mortar in 25 ml of saturated borax solution. The volume was adjusted to 100 ml with distilled water and boiled in a water bath for 15 min, and then cooled to room temperature. After being filtered, the filtrate (25 ml) was taken out and mixed with 4-aminobenzenesulfonic acid (2 ml, 4 g/L) and N-naphthyl-ethylenediamine dihydrochloride (1 ml, 2 g/L) and set aside for 15 min. The absorbance was measured at the wavelength of 538 nm, and the nitrite residual was expressed with the following formula: NaNO_2_ = (C ⋅ 2000)/(M ⋅ V). Here, C is the sodium nitrite concentration of the sample obtained from the calibration curve, M is the sample weight, and V is the volume of the extraction solution.

### TBARS

According to the method of Geeta and Yadav (2017) with slight modification, minced sausages (10 g) were homogenized with 50 ml of trichloroacetic acid solution (7.5% of trichloroacetic acid, 0.1% of EDTA, w/v), and then were filtered twice with three layers of filter paper. The filtrate was heated, and the distillate was collected. Then, 5 ml of the distillate and 5 ml of the TBA reagent (0.02 mol/L, thiobarbituric acid) were mixed and boiled in a water bath for 35 min. After being cooled, the absorbance was detected at 530 nm with a spectrophotometer (UV-1,200, MAPADA, Shanghai, China), and the TBA value was expressed with the formula below:

$$\mathrm{TBA}\,\mathrm{value}=\frac{50\times \left(\text{A}-\text{B}\right)}{\text{m}}$$

Here, A is the absorbance value of the sample solution, and B is the absorbance value of the blank.

### TVB-N

The measurement of the TVB-N content was carried out according to the method of Wang et al. (2016) with some modifications. The sausages were minced to small pieces and were homogenized in 90 ml of distilled water in a BagMixer for 90 s, stirred for 30 min, and filtered with three layers of filter paper. Then, 10 ml of the filtrate was mixed with 10 ml of MgO solution (10 g/L) and distilled for 5 min in semimicro Kjeldahl bottle. The distillate was mixed with 10 ml of boric acid solution (10 g/L) and titrated with 0.01 mol/L HCl. The content of the TVB-N was determined according to the consumption of hydrochloric acid.

### Amino Acid

The measurement of amino acid content was carried out by the ninhydrin colorimetric method. The sausages were minced to small pieces and homogenized in 90 ml of distilled water in a BagMixer for 90 s, boiled in a water bath for 10 min, cooled, and filtered. Then, 1 ml of the filtrate was mixed with 1 ml of citric acid buffer solution (0.2 mol/L, pH 6.86) and 1 ml of ninhydrin coloring solution in a test tube, and then the distilled water was added to make the total volume to 5.0 ml, shaken well, capped, and boiled in a water bath for 15 min. After being cooled, the absorbance was measured at 568 nm. Then, the content of amino acid was acquired by the standard curve. The standard curve was established through a series of concentrations of glycine standard solution (10, 20, 30, 40, and 50 μg/ml), and the processing steps were same as above.

### Met-Mb

The Met-Mb measurement was carried out according to our previous report (Zhu et al., 2020). Sausage samples (10 g) were homogenized in 25 ml of ice-cold phosphate buffer (0.01 mol/L, pH 6.8), placed in the dark at 4°C for 1 h, and then centrifuged at 4,000 g for 5 min at 4°C. Finally, the supernatant was filtered with a 0.45-μm nitrocellulose filter membrane. The Met-Mb content was determined by measuring the absorbance at 572, 562, 545, and 525 nm, respectively, using a spectrophotometer. The percentage of Met-Mb was determined with the formula below:

$$\text{Met}-\text{Mb}\%=\{-2.51\left(\frac{{\text{A}}_{572}}{{\text{A}}_{525}}\right)+0.777\left(\frac{{\text{A}}_{562}}{{\text{A}}_{525}}\right)+0.8\left(\frac{{\text{A}}_{545}}{{\text{A}}_{525}}\right)+1.098\}\times 100.$$

### Biogenic Amine

Sausage samples (1 g) were homogenized with 10 ml of trichloroacetic acid solution (5%, v/v) and extracted by ultrasonic extraction for 30 min. After centrifugation, the extraction (1 ml) was taken out and reacted with 0.2 ml of NaOH (2 mol/L) and 100 μl of benzoyl chloride at 40°C for 30 min. Then, the mixture was terminated with methanol and filtered with the membrane (0.22 μm) for high-performance liquid chromatography (HPLC) to determine the biogenic amine content. HPLC analysis was carried out with Syncronis C18 column according to our previous report (Zhu et al., 2020). The temperature of the column was set as 35°C with the injection volume of 20 μl and the UV detection wavelength of 254 nm. The gradient mobile phase contained solvent A and solvent B, and the procedure was started at a ratio of 70:30 (A:B) with a flow rate of 0.8 ml/min; subsequently, solvent B increased gradually to 70% within 38 min, and then maintained for 4 min.

### Sensory Analysis

Texture, flavor, slice, color, taste, sourness, and overall acceptability of the samples were evaluated according to a 10-point scale from 1 to 10 (Nassu et al., 2003). The evaluation was performed by 10 people who had experience in sensory evaluation of fermented sausages. The samples were sliced to 5-mm thickness and marked with three-digit random numbers. Then, the numbered slices were placed on white plates, and each sample was evaluated three times. Water and unsalted crackers were supplied to the panel to purify their palate between different samples.

### Statistical Analysis

All measurements were performed in triplicate, respectively. The data analysis was carried out using IBM SPSS Statistics 23.0 (IBM, New York, NY, United States). For multiple comparisons between more than three sample groups, the one-way analysis of variance (ANOVA) was applied and the significant difference between the groups were analyzed with Duncan’s multiple range tests. For the comparison between two sample groups, independent-sample *t-*test was employed. For both analysis methods, the significance was set at the level of *p* < 0.05.

## Results

### pH

**FIGURE 1:**
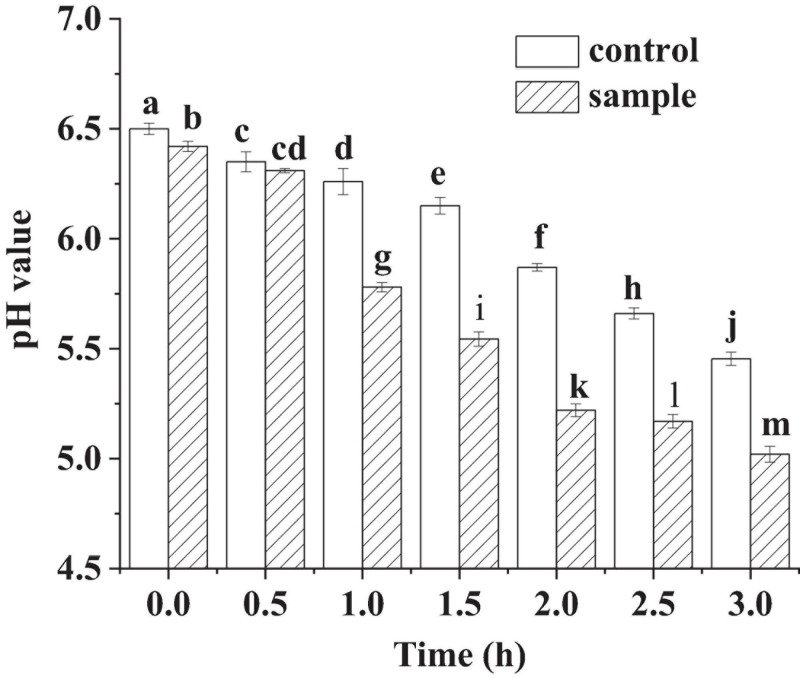
The pH of the sausages in fermentation, in which different lowercase letters meant that the pH was significantly different (*p* < 0.05).

pH is an important indicator to monitor the fermentation process of fermented sausages. In Figure 1, pH of the control and the sample showed very little decrease at the beginning of the fermentation (within 0.5 h). It might be because *L. fermentum* was in the growth phase of adapting to the new environment, resulting in low amount of lactic acid production. In the subsequent phase, the pH of the sample decreased sharply, which was much lower than that of the control. The sharp drop of the pH reflected that the anaerobic glycolysis increased and large amounts of lactic acid were produced in the growth of LAB (Chen et al., 2016). However, the pH of the sample was lower than the control during the whole fermentation process. For the sample sausages, when the pH dropped below 5.5, the speed of the pH decrease became slow. The phenomena might be attributed to the generation of non-protein nitrogen in the meat by proteolytic process, which would inhibit the decrease in pH through buffering the lactic acid (Chen et al., 2016). For the cathepsins will be activated and at pH 5.0–5.5 and accelerate the proteolysis (Visessanguan et al., 2003).

### Color Analysis

According to Table 1, the *L*^∗^ value of the sample and the control all decreased throughout the ripening process, and the value of the sample was lower than that of the control at the end of the 20th day. The *a*^∗^ value of the sample and the control was also affected by ripening time and declined over time. However, at the 20th day, no significant difference (*p <* 0.05) in *a*^∗^ value was found between the sample and the control, indicating that the strain inoculation could produce the pink color in the sausages nitrite-free and could make the color stable. This further indicated that the strain had a similar color development ability to nitrite. Another study also reported that LAB had the color formation ability in meat products nitrite-free (Gao et al., 2014). In addition, LAB application level was important for color development, and 10^8^ CFU/g inoculation of *L. fermentum* might develop the pink color similar to that produced by 60 mg/kg of nitrite (Zhang et al., 2007). The *b*^∗^ value of the sample and the control both increased during the ripening process, which indicated the oxidation of the sausages increased (Xiang et al., 2019). However, the value of the sample was much lower than that of the control in the whole ripening process, which indicated that the sample had good color and could inhibit lipid peroxidation (Xiang et al., 2019).

**TABLE 1:** The color of the sausages during ripening period.

	**Control**		**Sample**	
	**X**	**SD**	**X**	**SD**
L*				
0d	59.78^aA^	2.15	54.23^bA^	1.78
5d	54.34^aB^	1.73	50.45^aB^	3.12
10d	53.33^aB^	2.37	47.61^bBC^	2.16
15d	48.42^aC^	2.09	48.55^aBC^	1.40
20d	47.27^aC^	1.03	44.50^bC^	1.14
a*				
0d	12.34^aA^	1.14	10.71^aA^	0.67
5d	13.67^aA^	0.83	9.89^bA^	0.85
10d	7.63^aB^	0.45	6.38^aB^	0.83
15d	4.79^aC^	1.15	5.18^aBC^	0.48
20d	5.26^aC^	0.84	4.87^aC^	0.56
b*				
0d	1.25^aD^	0.31	0.44^bC^	0.08
5d	3.49^aC^	0.56	0.56^bBC^	0.11
10d	4.12^aC^	0.43	1.40^bB^	0.67
15d	5.25^aB^	0.21	3.16^bA^	0.78
20d	6.22^aA^	0.47	3.85^bA^	0.32

*The superscripted letters a b c represent significantly different (p < 0.05) in the same line, and A B C represent significantly different (p < 0.05) in the same column.*

### The Nitrite Content

**FIGURE 2:**
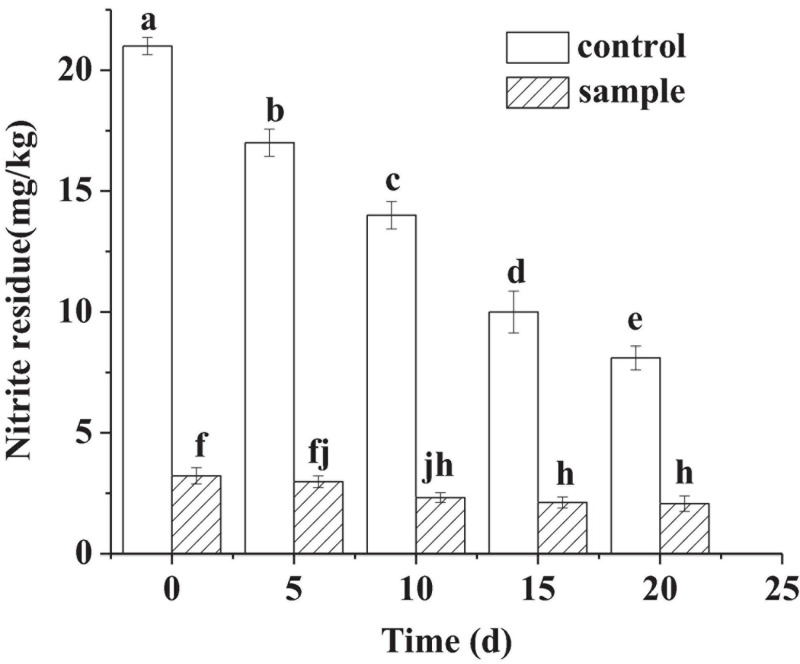
The nitrite content of the sausages during ripening period, in which different lowercase letters meant that nitrite content was significantly different (*p* < 0.05).

In Figure 2, although no nitrite was added to the sample, small amounts (3.22–2.07 mg/kg) of nitrite were detected in the sample sausages during the ripening process. In other study, small amounts of nitrite were also detected in the sausages with *L. fermentum* inoculation and nitrite free (Zhang et al., 2007). The source of nitrite in the sample was not clear, which might be derived from the curing process of meat. Zhang et al. (2007) regarded that the source of nitrite might be tap water, because it was very difficult to produce tap water of nitrite free. The nitrite content of the two groups decreased during the ripening process. The reduction in nitrite content in the control attributed to the reaction between the nitrite and myoglobin, which resulted in the production of nitrosomyoglobin (Mb-NO). For the sample, it might be that the strain had nitrite degradation ability according to the previous report (Yoo et al., 2015). However, the content of the sample was always significantly lower (*p* < 0.05) than that of the control during all the ripening process. On the 30th day, the content of the sample was 2.07 mg/kg, and that of the control was 8.1 mg/kg.

### TBARS

**FIGURE 3:**
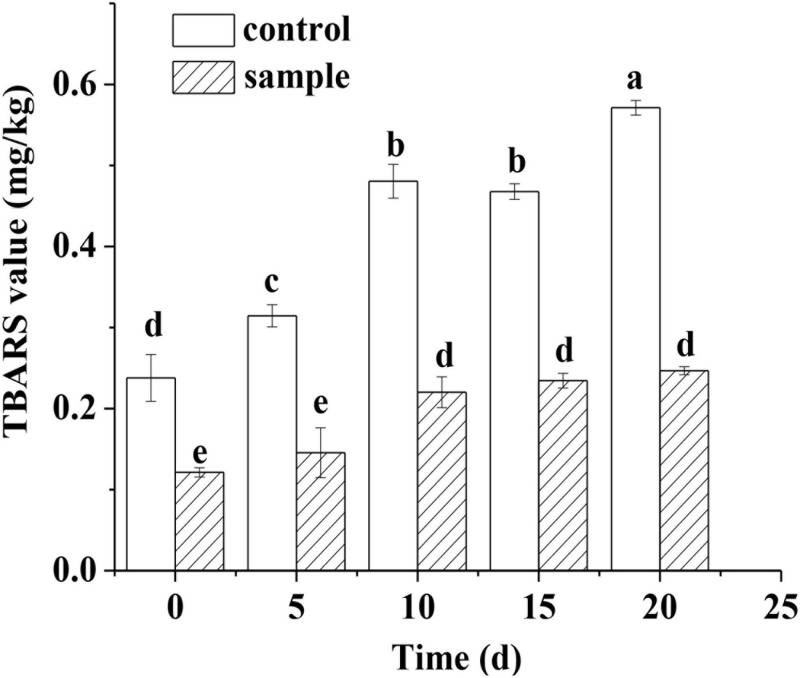
The TBARS content of the sausages during ripening period, in which different lowercase letters meant that TBARS content was significantly different (*p* < 0.05).

The TBA value reflects the extent of oxidation and rancidity of lipids in meat products. The TBA value of the two groups both increased constantly with extending ripening time (Figure 3). However, the TBA value of the control remained significantly higher (*p* < 0.05) than that of the sample during the whole ripening process (Figure 3). A previous study also had the similar result that the strain *Lactobacillus pentosus* inoculated in sausages had significantly inhibited the oxidation of lipids compared to the control (Sun et al., 2017). Although a previous report pointed out that nitrite could restrain the oxidation of lipids through different channels (Berardo et al., 2016), the result showed that the strain *L. fermentum* had stronger antioxidant capacity against lipids compared to the nitrite.

### TVB-N Content

**FIGURE 4:**
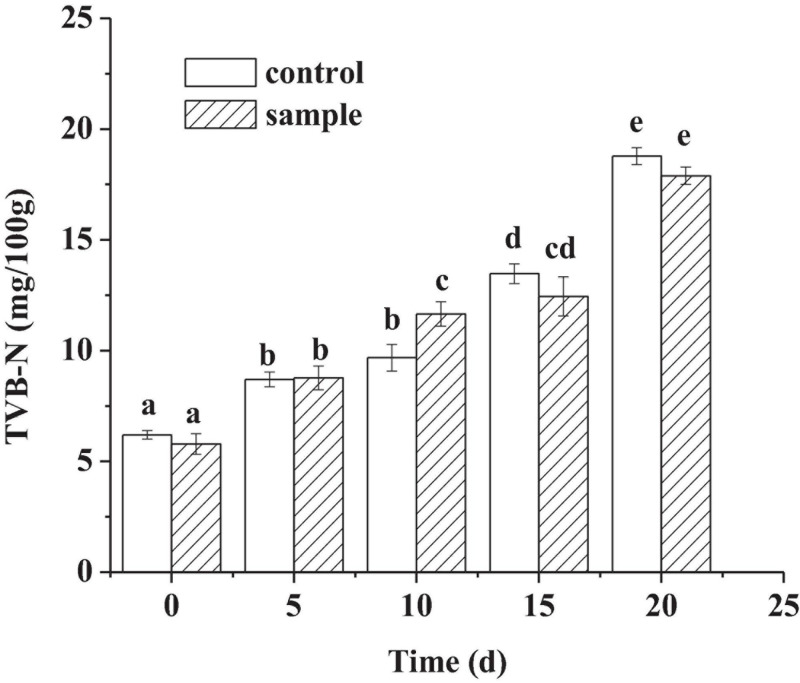
The TVB-N content of the sausages during ripening period, in which different lowercase letters meant that TVB-N content was significantly different (*p* < 0.05).

It could be seen from Figure 4 that the TVB-N content of the two groups increased continuously with time. TVB-N mainly reflects the degree of protein decomposition of meat products by microorganisms (Dabadé et al., 2015). Another study also regarded that TVB-N production might be due to the action of spoilage bacteria and endogenous enzymes (Song et al., 2011). There had been no significant difference *(p* < 0.05) between the sample and the control at the 20th day, indicating that the strain had a similar ability with that of nitrite in preventing protein decomposition from microorganisms and endogenous enzymes. Hence, the strain had a similar ability as nitrite to reduce protein decomposition and microbial risks.

### Free Amino Acid and Met-Mb

**FIGURE 5:**
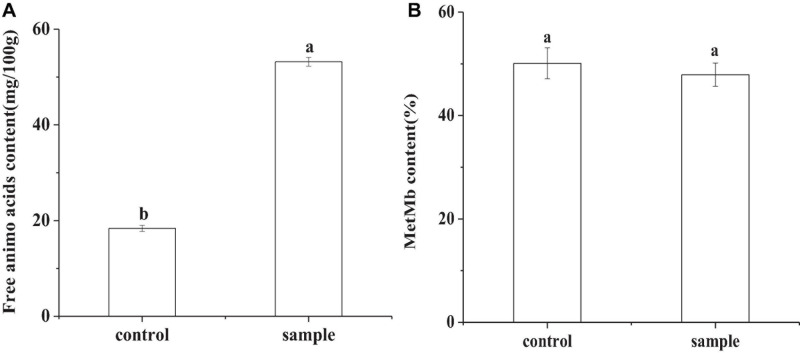
The free amino acid content **(A)** and the Met-Mb content **(B)** of the sausages at the end of ripening period, in which different lowercase letters meant that amino acid content or Met-Mb content was significantly different (*p* < 0.05).

In Figure 5A, the content of total amino acids in the control was 18.38 mg/100 g, while that of the sample was 53.19 mg/100 g. In fermented sausages, meat proteins are first hydrolyzed into polypeptides by endogenous proteases, and further hydrolyzed into amino acids by microbial protease (Hu et al., 2021). The higher total amino acid contents in the sample inoculated with LAB might due to the proteolytic activity of the strain (Hu et al., 2021). The highest total amino acid concentration was also observed in a previous study of the sausages inoculated with *L. plantarum* MLK 14-2 (Yoo et al., 2015). Met-Mb was the oxidation product of myoglobin in meat products, which had a negative effect on color (Howes et al., 2019). There had been no significant difference (*p* < 0.05) in the content of Met-Mb between the control and the sample (Figure 5B), which indicated that replacing nitrite with *L. fermentum* did not cause an increase in Met-Mb content.

### The Content of Biogenic Amines

Biogenic amines are usually found in a wide range of fermented foods and can bring health risks, in which histamine, tyramine, and phenethylamine are the primary cause of food poisoning. The European Union has established certain regulations on histamine with the maximum levels of 100 mg/kg (Sun et al., 2016). According to a previous report, the established maximum limits were 800 mg/kg for tyramine (Ercan et al., 2019) and 30 mg/kg for phenethylamine, respectively (Kandasamy et al., 2021). Seven kinds of biogenic amines including tyramine, putrescine, cadaverine, spermidine, tryptamine, phenylethylamine, and spermine were detected in the control (Table 2). For the sample, only tyramine, putrescine, cadaverine, spermidine, tryptamine, and spermine were detected, and no phenylethylamine was found. A previous report had suggested that the LAB used in the sausages might not contain phenylalanine decarboxylase activities, which resulted in no phenylethylamine produced (Pircher et al., 2007). Although the tyramine contents of sample was higher than the control, the contents of the two groups were all far below the specified limit (800 mg/kg). No histamine was detected both in the sample and the control, so the two group sausages had no histamine risk. In addition, the contents of spermidine and tryptamine decreased, and the contents of putrescine and cadaverine increased in the sample compared to the control. Hence, it is necessary to add other food additives together with the strain to inhibit the production of putrescine and cadaverine in future studies.

**TABLE 2:** The biogenic amine content of the sausages at the end of ripening period.

**Biogenic amine (mg/kg)**	**Sausage**	
	**Control**	**Sample**
Tyramine	78.24^b^	116.62^a^
Putrescine	6.60^b^	85.27^a^
Cadaverine	163.30^b^	462.13^a^
Spermidine	4.53^a^	2.50^b^
Tryptamine	5.88^a^	0.19^b^
Phenylethylamine	6.15	ND
Spermine	28.68^a^	31.21^a^
Histamine	ND	ND

*The superscripted letters a b c represent significantly different (p < 0.05) in the same line.*

### Sensory Analysis

**FIGURE 6:**
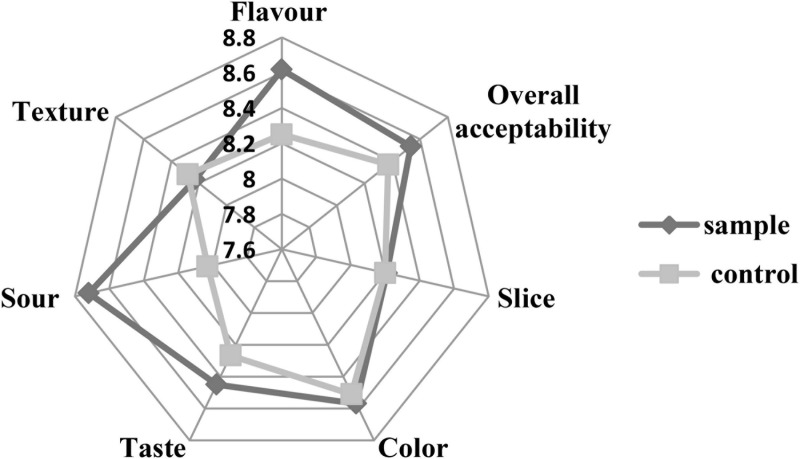
The sensory analysis of the sausages.

Sensory analysis was performed by evaluating the texture, flavor, slice, color, taste, sourness, and overall acceptability of the sausages. Figure 6 shows that the scores of the sample were similar to the control in the texture, slice, and color. The scores of taste, flavor, and sourness of the sample were better than those of the control. The relatively high acidity might be attributed to the production of lactic acid during fermentation of the LAB, which brought better and proper sourness (Odutayo et al., 2020). The better taste and flavor of the sample might be because the fermentation of the LAB could produce a variety of flavor components (Hu et al., 2019). Moreover, the inoculation of LAB could inhibit the lipid oxidation of the sausages, which prevents the unfavorite flavor (Hu et al., 2019). The result was consistent with the TBARS analysis (Figure 3) that the *L. fermentum* had stronger antioxidant capacity against lipid oxidation compared to the nitrite. Therefore, the overall acceptability of the sausages was better than the control, which was similar to a previous report that the LAB inoculation could make sausage quality better (Bachtarzi et al., 2020).

## Discussion

The low pH of the sample sausages fermented by *L. fermentum* (Figure 1) signified that the acidification of the sample was much stronger than that of the control fermented spontaneously (Wang et al., 2013). The pH sharp drop of the samples might induce conformational changes of proteins and result in acid-induced gelation (Zeng et al., 2013). In addition, low pH might decrease the risk of food-borne pathogens and improve the safety of the sausages (Gao et al., 2014), which would extend shelf life of the sausages (Sun et al., 2017). Because the growth of food borne pathogens and spoilage bacteria would be inhibited under acidic conditions, the spoilage of the sausage might be suppressed.

There had no significance in *a*^∗^ value (*p* > 0.05) between the sample and the control at the 20th day, which indicated that the *L. fermentum* had the color formation ability in meat products nitrite-free. The color formation ability might be because some LAB had nitric oxide synthase (NOS) activity, resulting in the production of NO, which reacted with myoglobin (Mb) in the inoculated sausages and generated the pink product of Mb-NO (Zhu et al., 2019). Compared to the control, the much lower *b*^∗^ value of the sample reflected that the strain inoculation could inhibit lipid peroxidation. The oxidation might result in the oxidation of Fe^2+^, which would cause the color of the sausages to appear brown (Ganhão et al., 2010). The color formation capacity and the antilipid oxidation ability of the strain indicated that the strain had the potential to replace nitrite for color development. In addition, nitrite can react with secondary amines to form nitrosamines (Sun et al., 2017), which has carcinogenic effects to humans. In this study, replacing nitrite with the *L. fermentum* significantly reduced the content of nitrite compared to the control (Figure 2). Therefore, replacing nitrite with the strain of *L. fermentum* decreased the residual of nitrite from the source and eliminate the carcinogenic risk of nitrite.

Lipid peroxidation will produce some harmful substances; therefore, the antioxidant function of the additives and the reduction of TBA value are very important (Gao et al., 2014). The strong antioxidant capacity of the strain *L. fermentum* against lipid oxidation (Figure 3) might be because the strain could scavenge free radical effectively (Chen et al., 2019). The sausage quality could be improved by preventing fat oxidation (Hu et al., 2019). In addition, the strain could inhibit the production of TVB-N (Figure 4), which is probably because the acidification process inhibited the growth of bacteriaceae and other bacteria (Sun et al., 2017). Because some microorganisms could secrete proteases, which might hydrolyze protein to produce nitrogen compounds including TVB-N (Sun et al., 2017).

The highest total amino acid concentration in the sample (Figure 5A) might be attributed to the strain hydrolyzed proteins forming the necessary amino acids required for growth (Hou et al., 2015). Amino acids play a key role in enhancing the flavor and increasing the nutrition of the sausages. The similar Met-Mb content in the control and the sample (Figure 5B) indicated that the strain *L. fermentum* had a similar ability as nitrite against Mb oxidation and avoided excessive production of Met-Mb. This ability might be due to the presence of Met-Mb reductase in the strain, which reduced Met-Mb to Mb and further produced Mb-NO (Zhu et al., 2019).

Small amounts of biogenic amines have important physiological effects on the human body, but if accumulated excessively, it would be life-threatening (Silla Santos, 1996). The results showed that substituting nitrite with the strain reduced the species of harmful biological amines and no phenylethylamine was detected in the sample (Table 2). Histamine is the most harmful amine and is closely related to human health problems (Nie et al., 2014). No histamine was detected in the sample, which might be because LAB could inhibit the growth of Enterobacteriaceae, which have been proven to be able to produce large amounts of histamine (Pircher et al., 2007). Even so, if other additives together with the strain were added to inhibit the production of tyramine, putrescine, and cadaverine, the biogenic amines risk could be reduced thoroughly, and the quality and safety of sausages would be greatly improved.

The quality of meat products depends on consumer’ appreciation and acceptance. Therefore, sensory analysis is very important for meat products according to the sensory evaluation test. The inoculation of the *L. fermentum* enhanced the taste, flavor, sourness, and overall acceptability of the sample sausages (Figure 6). The higher acidification of the sample could inhibit the growth of the pathogens and spoilage bacteria and make the LAB dominate the microflora (Xiao et al., 2020), which further enhanced the flavor of the sausages. On the other hand, the previous report indicated that there was a synergistic effect between sourness and saltiness, and the sourness enhanced the salty taste of the sausages, thereby giving the sausages a better taste (Hu et al., 2021). In addition, the strain has strong antioxidant capacity against lipid oxidation (Figure 3), which might prevent the rancid flavor of lipids oxidation.

## Conclusion

This study explored the positive effects of complete replacement of nitrite with the strain *L. fermentum* on the quality and safety of Chinese fermented sausages and evaluated the risk of this strain. The results revealed that the strain reduced the pH and the food-borne pathogens risk, and accelerated the acidification process and gelation formation of the sausages. In addition, the strain could produce the pink color similar to 50 mg/kg nitrite, significantly reducing the residual risk of nitrite in the sausages. In addition, the strain prevented fat oxidation, protein decomposition, and Mb oxidation and increased the free amino acid content. The strain reduced species of biogenic amines, and decreased the phenylethylamine, spermidine, and tryptamine risk. Some other additives that can inhibit the production of tyramine, putrescine, and cadaverine are necessary to replace nitrite with the strain *L. fermentum* together in the future research. Therefore, the strain has the potential to replace nitrite to produce healthier sausages.

## Data Availability Statement

The original contributions presented in the study are included in the article/supplementary material, further inquiries can be directed to the corresponding author/s.

## Author Contributions

YX was responsible for the method, data acquisition, curation, and analysis. YZ designed the study and drafted the original manuscript. Both authors contributed to the article and approved the submitted version.

## Conflict of Interest

The authors declare that the research was conducted in the absence of any commercial or financial relationships that could be construed as a potential conflict of interest.

## Publisher’s Note

All claims expressed in this article are solely those of the authors and do not necessarily represent those of their affiliated organizations, or those of the publisher, the editors and the reviewers. Any product that may be evaluated in this article, or claim that may be made by its manufacturer, is not guaranteed or endorsed by the publisher.
